# Port terminal mobile recognition based on combined YOLOv5s-DeepSort

**DOI:** 10.1371/journal.pone.0326376

**Published:** 2025-07-10

**Authors:** Chengzhi Wang, Donghong Chen, Zhen Liu, Yuanhao Li, Yifei Wang, Sanglan Zhao

**Affiliations:** School of River and Ocean Engineering, Chongqing Jiaotong University, Chongqing, China; University of Manitoba, CANADA

## Abstract

To solve the problem of reduced positioning accuracy caused by changes in scale, background and occlusion in port and dock video images, this research proposes an enhanced model combining YOLOv5s-DeepSORT, integrating target load recognition and trajectory tracking to improve adaptability to dock environments. The findings indicate that incorporating multi-scale convolution into YOLOv5s improved the robustness of multi-scale object detection, resulting in a 0.4% increase in mean Average Precision (mAP). Furthermore, the integration of an efficient pyramid segmentation attention (EPSA) network enhanced the accuracy of multi-scale feature fusion representation. The model’s mAP@0.5:0.95 increased by 1.2% following the introduction of EPSA. Finally, the original classification loss function was enhanced using a distributed sorting loss approach to mitigate the imbalance among loaded objects and the influence of background variations in the dock image sequence. This optimization led to a 3.1% improvement in multi-target tracking accuracy (MOTA). Experimental results on self-constructed datasets demonstrated an average accuracy of 90.9% and a detection accuracy of 92.2%, offering a valuable reference for target recognition and tracking in port and dock environments.

## 1 Introduction

In port terminal environments, target detection technology autonomously delivers dynamic semantic information and precise locations of targets via video imagery. This enables managers to effectively monitor moving objects [1–3], including ships, quay cranes, and trucks, across diverse terminal settings, thereby mitigating potential safety hazards. Prior research has focused on the spatial and temporal manipulation of objects within the workspace and their movements [4], with the aim of evaluating productivity or identifying potential risks [5].

The precision of multi-target tracking in surveillance video for real-time monitoring of dock loads is significantly contingent upon the accuracy of target detection. The tracking of transportation machinery across multiple cameras is predicated on the appearance and features of the machinery as captured by various cameras [6]. The identification and tracking of targets, along with their sequence and position across multiple non-overlapping camera views, have emerged as a research focus within the domains of computer vision and artificial intelligence. At present, within the domain of multi-camera target recognition and motion trajectory tracking [7], there is a need for further enhancement in the recognition accuracy and trajectory tracking precision of moving machinery. This necessity arises due to the diversity of camera viewpoints and the intricate environments characteristic of port terminals [8–10].

In recent years, target detection has emerged as a critical task in the field of computer vision, with the objective of identifying and localizing various objects within images or videos [11–13]. Presently, target detection techniques are primarily classified into region proposal-based methods, single-stage detectors, anchorless frame methods, and Transformer-based approaches. Among these, single-stage detectors are favored in both selection and practical applications due to their superior speed, efficiency, straightforward architecture and ease of implementation and deployment.

The YOLO (You Only Look Once) algorithm generates predictions directly from the input image, supports end-to-end training and is well-suited for real-time applications and resource-constrained devices [14,15]. It requires minimal hyperparameter tuning, and its modern iterations demonstrate superior performance in detecting small targets and managing dense scenes [16]. Han et al. [17] enhanced this framework by integrating EfficientVit to replace the original backbone network, employing efficiently decoupled detection heads to separately address classification and regression tasks. Additionally, they combined the Complete Intersection over Union (CIoU) loss with the Normalized Wasserstein Distance (NWD) loss for localization, resulting in a 9.6% improvement in detection accuracy, however, there remains potential for enhancement in real-time capability and detection speed. Ren et al. [18] introduce a lightweight detection algorithm that incorporates a Distributed Shift Convolution (DSConv) layer and a Compressed Excitation (SE) attention mechanism. This approach replaces certain original convolutional and C3 modules and employs skip connections for multi-scale feature fusion within a Ghost module. The modified model achieves a 1.0% increase in average accuracy compared to the original algorithm. Nevertheless, the focus on achieving a lightweight model has overlooked the implications for detection speed. Li et al. [19] introduced a CAC3 module incorporating an attention mechanism to enhance the neck BiFPN feature pyramid network. Experimental results demonstrated that mAP of the improved model in detecting trapped individuals of varying proportions and postures increased by 7.4% and 24.9%, respectively, compared to the YOLOv5s model. However, the model’s robustness against interference remains an area for improvement. Wang et al. [20] addressed the issue of mismatch between the detector’s receptive field and the target object by integrating a receptive field extension block into the backbone network. Simultaneously, the integration of the target detection head and the attention mechanism within the scale-aware feature layer resulted in a coherent enhancement, leading to a 6.6% improvement in average detection accuracy and an augmented predictive capability of the model. However, the detection speed remains suboptimal and requires further enhancement. Consequently, future research should prioritize both detection accuracy and speed to identify and develop more effective improvement methodologies.

Multiple Object Tracking (MOT) [21] aims to monitor numerous targets concurrently within a video sequence. Presently, widely adopted multi-target tracking methodologies encompass Tracking-by-Detection, Joint Detection and Tracking, and Deep Learning-based approaches. Among these, DeepSORT demonstrates superior performance in multi-target tracking, attributed to its high-precision target association and its capability to leverage deep learning features, thereby enhancing the robustness and adaptability of tracking. DeepSORT is well-suited for continuous tracking demands in complex environments [22,23] and exhibits high scalability, allowing for seamless integration with various target detection algorithms.

Huang et al. [24] proposed an online tracking method that integrates a five-frame difference approach with the Deep SORT algorithm to address the challenge of tracking spatial dynamic targets, including those experiencing occlusion. This method aims to facilitate the recognition of dynamic targets prior to tracking. While the algorithm demonstrates high effectiveness and superiority under conditions of strong illumination and occlusion, it falls short in tracking occlusion and attitude changes across all scenarios, thereby limiting its utility for comprehensive spatial capturing. In a related study, Yang et al. [25] modified the re-identification network structure of DeepSort. Using a Multi-Task Learning (MTL) framework, the foreign object tracking algorithm of DeepSort was substituted with an Object-Specific Attention (OSA) module, resulting in improvements of 6% in MOTA and 3.9% in Multiple Object Tracking Precision (MOTP). However, the enhanced algorithm encountered limitations in its application range and required further improvements in usability. Thioanh Bui et al. [26] enhanced the Kalman Filter (KF) algorithm within the DeepSORT framework by substituting the aspect ratio of the vehicle prediction box with its width in the original KF algorithm. Additionally, they replaced the conventional convolutional neural network (CNN) model with an improved ResNet36 architecture as the backbone network for feature extraction in the DeepSort re-identification network. Despite these advancements, the algorithm continues to exhibit certain inherent limitations, including inaccuracies in localizing target boundaries and suboptimal performance in multi-scale target detection. Luo et al. [27] modified the architecture of the DeepSORT appearance feature extraction network and subsequently retrained it using a vehicle re-identification dataset. However, the accuracy of this approach is influenced by several factors that could be mitigated through the application of an image enhancement optimization algorithm or by increasing video resolution. Consequently, future research should focus on enhancing the robustness and environmental applicability of multi-target tracking systems.

Considering the aforementioned challenges, the YOLOv5s model has been selected as the foundational model for this research due to its optimal balance between speed and accuracy, making it particularly suitable for real-time target recognition and tracking in port terminal environments. The primary contributions of this research are threefold: (Ⅰ) the collection and annotation of domestic and international dock images to create a comprehensive dataset; (Ⅱ) the integration of Pointwise Separable Convolution (PSConv) and EPSA mechanisms to enhance the accuracy of target detection in complex scenarios; and (Ⅲ) the integration of the GhostNet module and the DIoU metrics aims to reduce computational complexity and enhance the efficiency and accuracy of target tracking within video sequences.

## 2 Improved YOLOv5s-DeepSORT Port Terminal Target Detection Tracking Algorithm

### 2.1 Preparation of target data for port terminals

**Fig 1:**
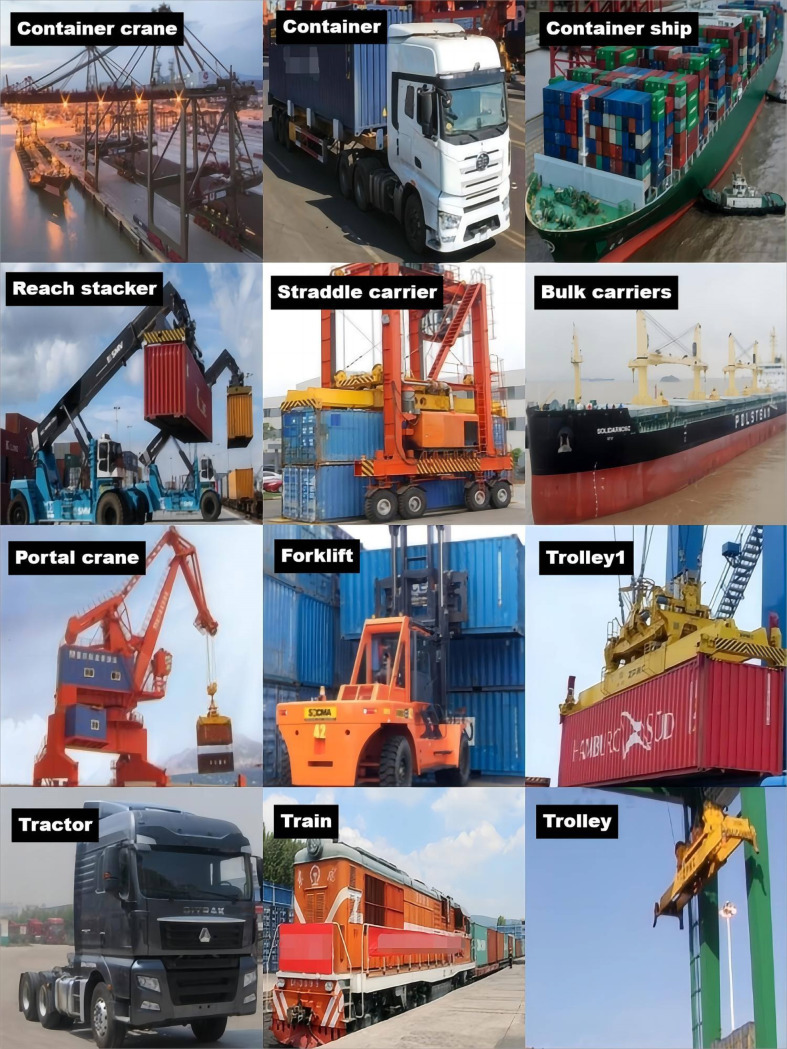
Overview of the dock target datasets.

For the development of an object detection model based on YOLO, the establishment of a comprehensive dataset is imperative. However, the SeaShips Dataset comprises only six ship categories, which, while sufficient for ship detection and classification tasks, fails to address the comprehensive requirements of port operational scenarios. It lacks annotations and diversity for detecting and tracking complex operational machinery critical to daily port logistics, such as trolley, container trains, and Forklift. This study develops a novel port terminal dataset to address the evolving dynamics of port operations by utilizing both on-site and web-sourced images (Fig 1). The images in this dataset are derived from on-site shooting at the Laotangshan Wharf in Zhoushan City, Zhejiang Province, as well as from browsers such as IE, Baidu, and Sohu. Mainly, direct and concentrated searches are carried out for twelve types of images, including container loading and unloading bridges, container front-end handling vehicles, portal cranes, tractors, container trucks, straddle carriers, container forklifts, port trains, container ships, bulk carriers, container trolleys of gantry cranes, and trolleys of gantry cranes. The collection and analysis method complied with the terms and conditions for the source of the data. The dataset comprises 5134 photographs depicting targets within dock scenes, which span diverse types of docks, varying camera angles, different resolutions, and target distance variations. Additionally, the dataset includes several detection challenges such as crowded scenes, occlusions, and multi-scale targets, thereby providing comprehensive reference samples for target identification and analysis.

Following the collection of photographs, the image data were meticulously annotated utilizing Labelimg software. The surveillance image dataset samples were predominantly labeled with the x and y coordinates of the target image’s center of mass, alongside the width and height dimensions of the bounding box. Concurrently, to enhance the model’s generalization capability, image augmentation techniques were employed during the training phase to increase sample diversity. The augmentation methods implemented in this study comprised random rotation, cropping, flipping, and affine transformation. Furthermore, an additional test dataset was created, encompassing surveillance images with the three most prevalent resolutions (1080×960 pixels, 1280×720 pixels and 1920×1080 pixels).

### 2.2 Optimization of target detection networks

#### 2.2.1 Introduction of multi-scale convolution in PANet.

To enhance the model’s robustness in handling multi-scale targets, multi-scale convolution was incorporated into the PANet network structure of YOLOv5s following extensive preliminary experiments. In the dock scene, the load objects display a range of sizes and shapes, accompanied by complex interactive motions. Consequently, the detection model must enhance its capability to manage the scale variation of these load objects. The benchmark YOLOv5s model primarily employs a regular convolution kernel with a fixed shape to extract information from targets of varying scales across different-sized feature maps. However, the receptive field of this convolution kernel is limited. Additionally, increasing the size of the convolution kernel results in a higher number of parameters. Simultaneously, increasing the size of the convolutional kernel results in a higher number of parameters and greater computational cost. To enhance the efficiency of convolutional networks, researchers have proposed several significant methods, including dilated convolution (DConv) [28], depthwise separable convolution (DSConv) [29], grouped convolution (GConv) [30] and mixed depth convolution (MixConv) [31], among others. Nevertheless, these generalized CNNs typically employ a single-sized convolutional kernel, which constrains their effectiveness in processing multi-scale features. To achieve multi-scale computation of feature maps on channels with finer granularity without incurring additional computational costs, the multi-scale convolution PSConv, utilizing a single convolution kernel, has been introduced. In a standard convolutional layer, the convolution operation is typically defined as follows:


$$H_{c,x,y}=\sum_{k=1}^{c_{in}}\sum_{i=-\frac{k-1}{2}}^{\frac{k-1}{2}}\sum_{j=-\frac{k-1}{2}}^{\frac{k-1}{2}}f_{c,k,i,j}F_{k,x+i,y+j}$$

Which $F\in R^{C_{in}\times H\times W}$ denotes the input feature map $\left(C_{in}\times H\times W\right)$, $C_{in}$ is the number of input channels, $f_{c,k,i,j}$ is the convolution filter and $H_{c,x,y}$ is the output feature map.Furthermore, the expansion convolution with expansion rate d can be expressed as follows:


$$H_{c,x,y}=\sum_{k=1}^{c_{in}}\sum_{i=-\frac{k-1}{2}}^{\frac{k-1}{2}}\sum_{j=-\frac{k-1}{2}}^{\frac{k-1}{2}}f_{c,k,i,j}F_{k,x+id,y+jd}$$

PSConv integrates various expansion rates to extract multi-scale features, with these expansion rates being allocated to the convolution kernel in the following manner:


$$H_{c,x,y}=\sum_{k=1}^{c_{in}}\sum_{i=-\frac{k-1}{2}}^{\frac{k-1}{2}}\sum_{j=-\frac{k-1}{2}}^{\frac{k-1}{2}}f_{c,k,i,j}F_{k,x+iD_{\left(c,k\right)},y+jD_{\left(C,k\right)}}$$

The fixed nature of conventional convolution in channel YOLOv5s results in a receptive field on the feature map that lacks the flexibility afforded by multi-scale convolution. Given that the incorporation of multi-scale convolution within the residual network of ResNet50 has been shown to significantly enhance model accuracy, this study proposes the integration of multi-scale convolution into the PANet network structure of channel YOLOv5s, thereby replacing some of the conventional convolution layers.

#### 2.2.2 Improved EPSA feature fusion network.

Following the operations detailed in the previous section, the objective of this study was to further enhance the detection performance of the model on the self-checking dataset by applying an attention mechanism to the feature vector extraction process. This approach is intended to improve the representation of critical information within the feature vectors. Recent advancements, such as Squeeze-and-Excitation Networks(SENet) [32], Convolutional Block Attention Module(CBAM) [33,34] and Efficient Channel Attention for Deep Convolutional Neural Networks(ECANet) [35,36], have significantly improved model performance; however, their methods for handling feature map scales are limited to a singular approach. In contrast, the EPSA mechanism is more adept at managing multi-scale input tensors. Therefore, in this study, the EPSA structure was integrated into the YOLOv5s model.

Initially, the Pyramid Segmentation Attention (PSA) method partitions the input tensor into S segments, each of which is processed through a distinct Statistical process control (SPC) module. These modules progressively expand the convolution kernel for each segment, employing kernel sizes such as k = 3, 5, 7, and 9. Following convolution with these varying kernel sizes, the output tensors from each segment are concatenated along the channel dimension. Furthermore, PSA incorporates the Squeeze-and-Excitation (SE) weighting mechanism to refine the output of the SPC module, thereby obtaining the attention weights of the feature maps across different scales.

**Fig 2:**
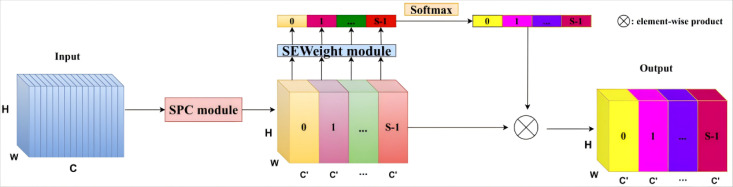
Structure of the EPSA Module [37].


As shown in Fig 2, this strategy not only integrates contextual information across multiple levels but also enhances pixel-level attention. The final output is produced following the recalibration of weights, which are normalized using a SoftMax function and subsequently applied to the output of the SPC module.

$$g_{c}=\frac{1}{H\times W}\sum_{i=1}^{H}\sum_{j=1}^{W}x_{c}\left(i,j\right)$$

$$AM=softmax(z_{i})$$

To extract more prominent features from the deep information of the loaded object images, the lightweight attention module of EPSA is introduced. Additionally, a PANet multi-scale convolutional network is embedded within Yolov5s to recalibrate the feature channels.

### 2.3 Optimization of port terminal target tracking model based on Deep SORT

#### 2.3.1 Epigenetic feature extraction network construction based on GhostNET module.

**Fig 3:**
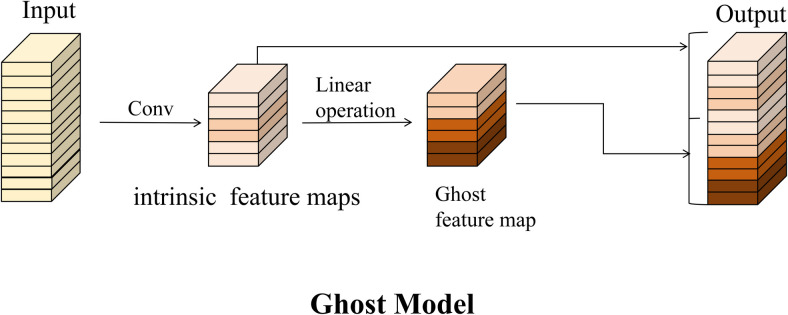
Ghost Module.

To mitigate the complexity of the feature extraction network and efficiently decrease the computational load associated with floating-point arithmetic, this study incorporates the Ghost module (Fig 3) into the structural design of the apparent feature network. The original epigenetic feature extraction network comprises multiple convolutional layers with 128, 64 and 32 channels, necessitating substantial memory and computational resources for conventional convolution operations across these channels. In this study, the Ghost module is incorporated into the feature extraction network. This module initially produces a subset of intrinsic feature maps derived from the original feature maps. Subsequently, it generates new feature maps through a linear transformation operation utilizing 3x3 convolution on these intrinsic feature maps. Specifically, the network first creates intrinsic feature maps via conventional convolution at the specified channel locations of the original feature maps. Following this, new Ghost feature maps are generated through the linear transformation operation of 3x3 convolution on the intrinsic feature maps, and the two segments of the feature maps are subsequently concatenated along the channel dimension, and the final output is derived by summing the inputs from the two Ghost modules. Given that the original feature extraction network of DeepSort is structured around a wide residual network, the Ghost module is integrated by embedding it within the base module of the 6-layer residual network, specifically from Residual4 to Residual9, thereby replacing the standard 2D convolutional layers. In configuring the network architecture, it is noteworthy that the original network employs a Maxpool layer at the third layer to extract key feature information. In this study, adaptive max pooling was used instead of the original pooling layer, as shown in Table 1.

**Table 1:** Feature Extraction Network Architecture.

Designation	Array size	Loop step size	Function output size
Conv1	3×3	1	32×128×64
Conv2	3×3	1	32×128×64
Max Pool3	3×3	2	32×64×32
Residual4	3×3	1	32×64×32
Residual5	3×3	1	32×64×32
Residual6	3×3	2	64×32×16
Residual7	3×3	1	64×32×16
Residual8	3×3	2	128×16×8
Residual9	3×3	1	128×16×8
Dense			128
Normalization			128

#### 2.3.2 Optimization of association matching module based on distance and intersection ratio.

To enhance the optimization of the matching effect within the target tracking algorithm, the Distance Intersection over Union (DIoU) metric is integrated into the re-matching module.

The DIoU metric, which is detailed in Equation 6, offers a more comprehensive evaluation by incorporating the distance between the centers of the bounding boxes, thereby providing a more robust assessment compared to other metrics. In this context, d represents the Euclidean distance between the central positions of the prediction frame and the detection frame. The variable c signifies the diagonal distance of the smallest enclosing matrix that encompasses the intersection of the prediction frame and the detection frame.


$$DIoU=IoU-\frac{d^{2}}{c^{2}}$$

**Fig 4:**
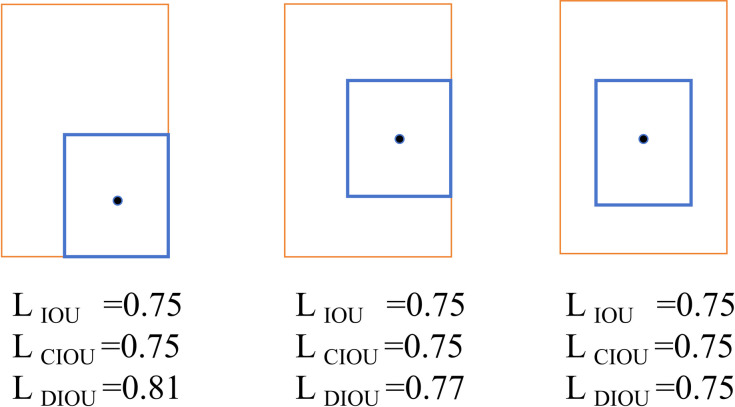
Examples of calculation of IoU, GIoU and DIoU metrics.

Based on the definitions of Intersection over Union (IoU), Generalized Intersection over Union (GIoU) and Distance Intersection over Union (DIoU), Fig 4 illustrates an example demonstrating the calculation results of these three indices when both the prediction box and the detection box are included. In the scenario where one box is encompassed by the prediction box (orange box) and the detection box (blue box), the values of IoU and GIoU are 0.75. However, the values of DIoU are 0.81 and 0.77, respectively, indicating that DIoU provides a more accurate assessment of the matching situation compared to IoU and GIoU. The proposed method demonstrates superiority over IoU and GIoU by providing a more precise assessment of the matching situation. By incorporating distance metrics, this approach more effectively captures the positional relationship between two bounding boxes compared to IoU and GIoU. Consequently, it yields improved matching accuracy, as evidenced by performance metrics of 0.81 and 0.77, which further underscore its capacity to reflect the spatial relationship between two frames more accurately than traditional methods. To enhance the efficacy of the matching process, this paper introduces the DIoU as a metric for comparing detected frames with predicted frames during correlation matching.

### 2.4 Overall structure

**Fig 5:**
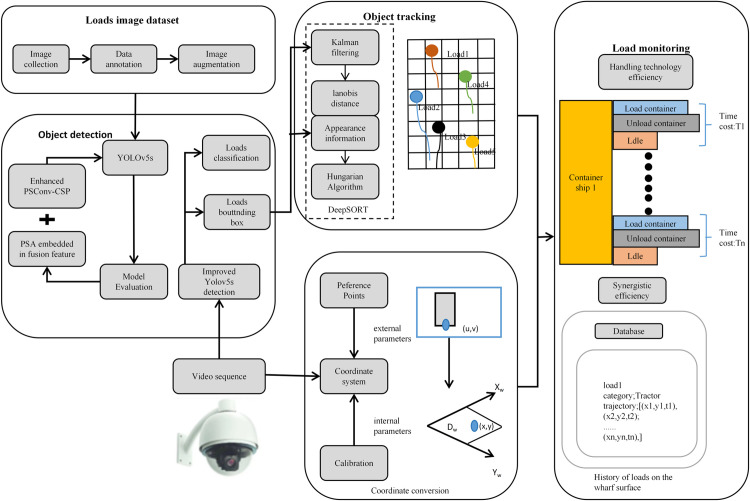
An Overall Framework for Load Target Detection and Recognition.

Utilizing the acquired video footage of the dock, the type of moving target is identified using the target detection method outlined in the previous study. Subsequently, trajectory tracking techniques are employed to ascertain the activity information of the moving target. The methodology involves enhancing Yolov5s for video stream access, utilizing OpenCV for video loading, and processing the incoming video stream. The video frames are resized to a standard dimension and subsequently fed into the target detection network. The real-time processed frames are then rendered in a display window, which exhibits the bounding box coordinates and category confidence scores for various harbor moving targets. This approach facilitates the rapid identification of the location and type of machinery. Combining the DeepSORT algorithm, configuring the parameters of the tracker and detector, assigning a unique identification ID to each target in the object detection module, and tracking moving targets in real-time online. The identification information of all targets in the video can be recorded in the data file, making it convenient to query the motion trajectory and quantity of load targets online. For surveillance videos of entrances and exits, information such as their movement speed can also be estimated, as described in Fig 5.

## 3 Model training and selection of evaluation indicators

### 3.1 Experimental environment and model training

#### (i) Target detection.

The proposed method utilizes PyTorch, a deep learning framework. All experiments were conducted on a server featuring an Intel(R) Xeon(R) CPU E5-2637 V4, an NVIDIA RTX 2080Ti GPU with 11GB of graphics memory, and the Ubuntu 16.04 operating system.

The dataset was partitioned into training and test sets with a ratio of 9:1, and the images were resized to 640 × 640 pixels prior to training. The learning rate, momentum, and weight decay were configured to 0.001, 0.9, and 0.0001, respectively. Each training batch comprised 32 images, and the model was trained to 300 epochs. Pre-trained weights were employed for initialization.

**Fig 6:**
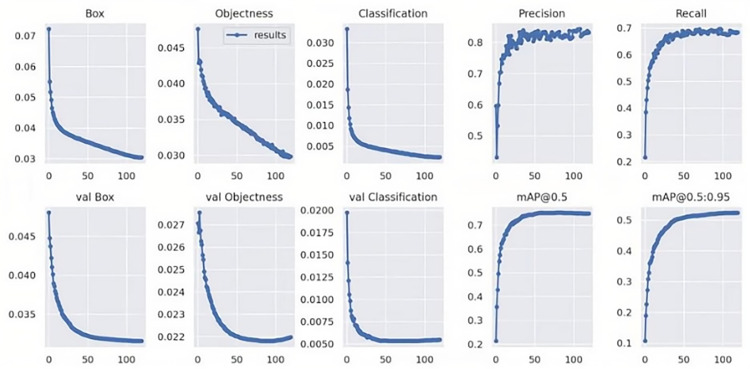
Model training process.

The training outcomes are illustrated in Fig 6.

**Fig 7:**
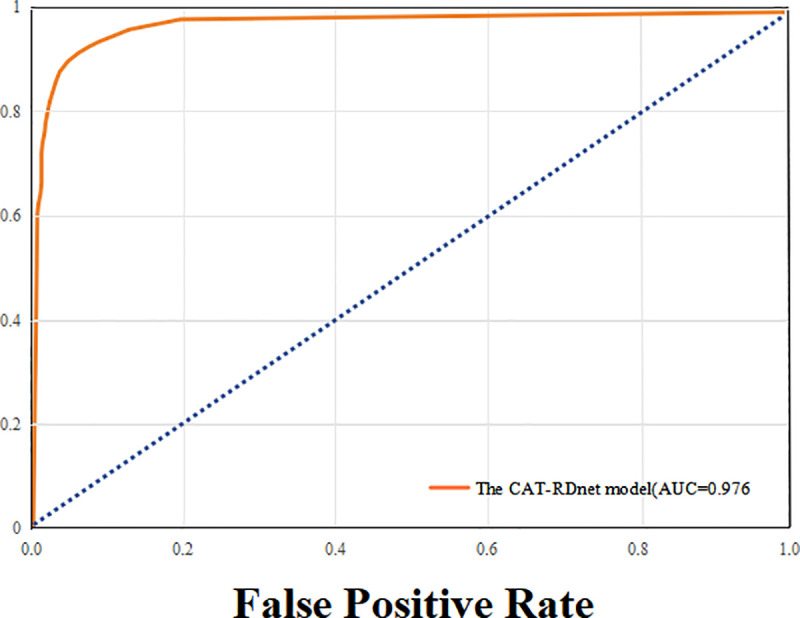
ROC curve.

The Receiver Operating Characteristic (ROC) curve, derived from the classification results on the test dataset, is presented in Fig 7.

#### (ii) Target tracking.

Regarding the training evaluation of the feature extraction network, the parameters are as follows: the number of training epochs is set to 50, the initial learning rate is 0.1, the batch size is 64, the momentum is configured to 0.9, weight decay is applied at a rate of 5e^-4^, the cross-entropy function is utilized as the loss function, and Stochastic Gradient Descent (SGD) is employed as the optimizer. The parameters for the DeepSORT algorithm have been configured as follows: the maximum distance is set to 0.2, the minimum confidence level to 0.3, the non-maximum suppression threshold to 0.5, the maximum Intersection over Union (IoU) distance to 0.7, the maximum age to 70 frames, and the maximum feature extraction range to 100 units.

**Fig 8:**
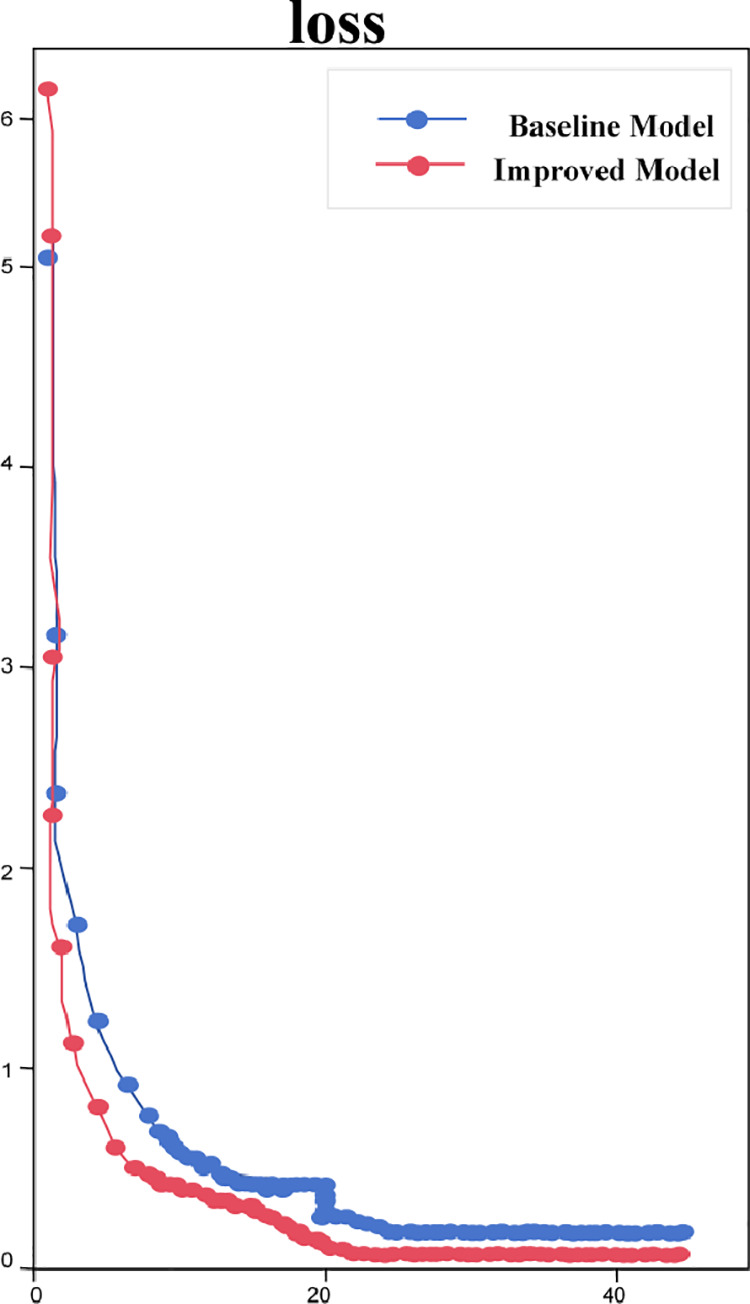
Convergence states for model training losses.

During the training process, the loss function values of the network are recorded by rounds, and it can be seen from Fig 8 that both the original model and the model constructed based on the GhostNet module show a gradual decreasing trend during the training process, and the loss function of the original model eventually stabilizes at about 0.2 during the training process, whereas the improved model ultimately stabilizes and converges at about 0.1. This demonstrates that the enhanced model not only increases re-recognition accuracy but also exhibits reduced curve fluctuation during the training process compared to the original model. This reduction in fluctuation suggests that the enhanced model decreases computational complexity, facilitates the convergence of the loss function and contributes to greater network stability throughout training.

### 3.2 Selection of evaluation indicators

#### (i) Target detection.

Precision, recall, Average Precision (AP) and mAP metrics were selected to assess the performance of the proposed model.


$$Precision=\frac{TP}{TP+FP}$$

$$Recall=\frac{TP}{TP+FN}$$

$$AP=\int_{0}^{1}P(R)dR$$

$$mAP=\frac{\sum_{i=1}^{n}AP_{i}}{n}$$

In this context, TP (true positive) represents the number of detections where the IoU between the predicted and actual bounding boxes exceeds 0.5. FP (false positive) indicates the number of incorrectly detected objects with an IoU less than 0.5, while FN (false negative) corresponds to the number of objects that were not detected. The P(R) curve illustrates the relationship between precision and recall. Here, n denotes the total number of classes and ${AP}_{i}$ represents the Average Precision for class i.

#### (ii) Target tracking.

In this study, algorithmic performance evaluation metrics for target tracking include the commonly utilized Multi-target Tracking Accuracy (MOTA) and Multi-target Tracking Precision (MOTP), as well as ID Switch (IDSW), Identification Precision (IDP) and Identification Recall (IDR).


$$MOTA=1-\frac{\sum \left(FN+FP+IDSW\right)}{\sum GT}$$

In the aforementioned equation pertaining to mechanical target tracking, FP (False Positive) denotes instances where the prediction frame of the Kalman filter for the mechanical target does not correctly correspond to the detection frame. FN (False Negative) signifies cases where the prediction frame of the Kalman filter fails to align with the detection frame. GT (Ground Truth) represents the actual trajectory information. ID Switch (IDSW) refers to the number of occurrences where the identifier associated with the real trajectory has been altered.


$$MOTP=\frac{\sum_{t,i}d_{t,i}}{\sum_{t}c_{t}}$$

Where $c_{t}$ denotes the number of tracking matches in frame t and $d_{t,i}$ denotes the error between matching pairs. This part also focuses on the metrics for tracking IDs of moving targets in the dock, so the IDP and IDR on identity localization are introduced as metrics.


$$IDP=\frac{IDTP}{IDTP+IDFP}$$

$$IDR=\frac{IDTP}{IDTP+IDFN}$$

where IDTP denotes the number of IDs for true cases, IDFP denotes the number of IDs for false positive cases and IDFN denotes the number of IDs for false negative cases.

## 4 Results and discussion

### 4.1 Evaluation of target detection model results

#### (i) Model comparisons.

**Fig 9:**
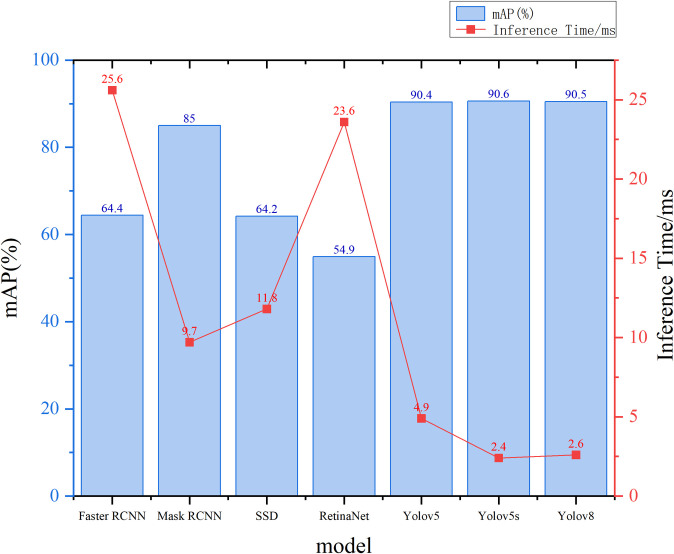
Detection accuracy of different models.

To preliminarily assess the accuracy of various models prior to experimentation, Faster Region-based Convolutional Neural Networks (Faster R-CNN), Single Shot Multibox Detector (SSD), Retina Neural Network (RetinaNet), Mask RCNN, YOLOv8s and YOLOv5s models were trained. The primary categories of trained models encompass Container, Truck, Crane, Bulk, Carriers, Forklift, Reach Stacker, Ship, Straddle Carrier, Train, Trolley, Trolley1 and Tractor. As illustrated in Fig 9, the YOLOv5s model demonstrated a mAP of 90.6%, surpassing other models. Concurrently, its inference time is 2.4 milliseconds, which is 23.2 milliseconds, 9.4 milliseconds, 21.2 milliseconds,7.3 milliseconds and 0.2 millisecond faster than the inference times of the Faster R-CNN, SSD, RetinaNet, Mask RCNN and YOLOv8s models, respectively. This performance meets the real-time detection requirements of the dock site. Consequently, the YOLOv5s model, which proposed in this research, method excels in balancing accuracy and speed for monitoring image datasets, outperforming other existing methods.

#### (ii) Validation of results.

As shown in Table 2, this study conducted a comprehensive comparison of the detection results obtained from the enhanced model against those of other existing models. Utilizing YOLOv5s as the baseline, the integration of PSConv resulted in a mAP@0.5 reaching 91.0%, accompanied by an average processing time of 2.6 milliseconds. It is noteworthy that the incorporation of additional parameters within the PSConv unit led to a marginal increase in processing time, specifically 0.2 milliseconds longer than the baseline model. Furthermore, the introduction of the EPSA yielded an increase in Precision from 87.6% to 89.1.%. Additionally, it was observed that the fully improved model showed improvements were observed in mAP@0.5:0.95, Recall, and F1 scores, which increased by 1.4%, 1.8% and 0.8%, respectively, when compared to the baseline model.

As presented in Table 2 , although the model incorporating only PSConv exhibits a highermAP@0.5, this metric is evaluated at a lower IoU threshold (0.5), which is lenient and does notcomprehensively represent the model's localization accuracy. In contrast, the enhanced model performs better in mAP@0.5:0.95, attaining a high average precision across multiple IoU thresholds ranging from 0.5 to 0.95, suggesting that its detection capabilities are more reliable under rigorous conditions. It is more applicable to the requirements of small object detection. Additionally, the enhanced model surpasses the PSConv-only model in terms of Precision, Recall, and F1 score, indicating that it not only captures more targets but also reduces false positives, demonstrating a better balance in positive and negative sample classification. Despite the marginally slower inference speed of the enhanced model, the demand for high accuracy typically outweighs the requirement for speed in port target detection tasks. Therefore, considering metrics such as mAP@0.5:0.95, Precision, Recall, and F1 score, the inclusion of EPSA is indispensable, and the overall performance of the enhanced model is better adapted for tasks in port environments.

**Table 2:** Detection accuracy of different models.

Model	mAP @0.5 (%)	mAP@0.5:0.95 (%)	Precision (%)	Recall (%)	F1 (%)	Inference Time/ms
Yolo	90.6	74.2	89.7	87.6	88.6	2.4
Yolo+ PSConv	91.0	75.4	90.7	85.5	85.5	2.6
Yolo+EPSA	90.05	74.4	92.9	89.1	89.1	3.6
Yolo+PSConv+EPSA	90.9	75.6	92.2	89.4	89.4	3.7

**Fig 10:**
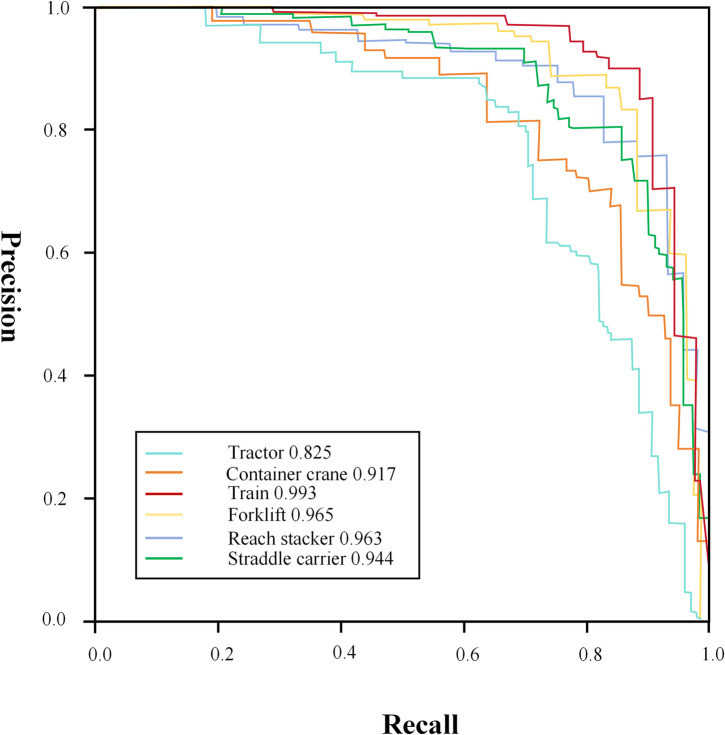
PR curves for the improved models.

**Fig 11:**
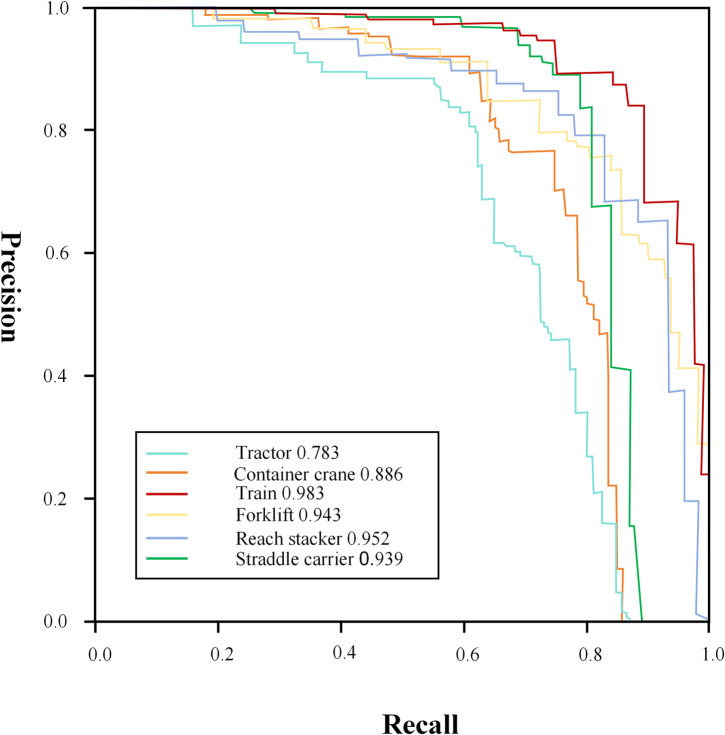
PR curves for the original models.

To demonstrate the efficacy of the proposed model, this study analyzed the accuracy and recall metrics for the recognition of six distinct dock machinery behaviors, as illustrated in Fig 10. The results indicate that the algorithm is capable of accurately identifying each dock machinery behavior, thereby achieving the anticipated outcomes. Specifically, the improved model exhibits higher recognition accuracies for Train, Reach Stacker, and Forklift, with values of 99.3%, 96.3% and 96.5%, respectively. However, the recognition accuracy for Tractors is comparatively lower, at 82.5%. In the PR curves, the majority of targets exhibit comparable accuracy results. Notably, compared to Fig 11, the enhanced model demonstrates a significant improvement in detection accuracy for Tractors and Cranes, with increases of 4.0% and 3.0%, respectively, over the original model. This suggests that the enhanced model provides a targeted improvement in detection accuracy for dock-related targets.

**Fig 12:**
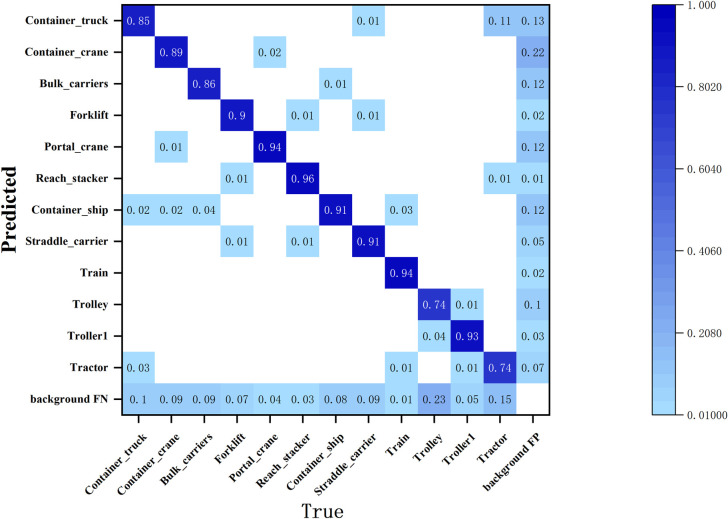
Confusion matrix for the original models.

**Fig 13:**
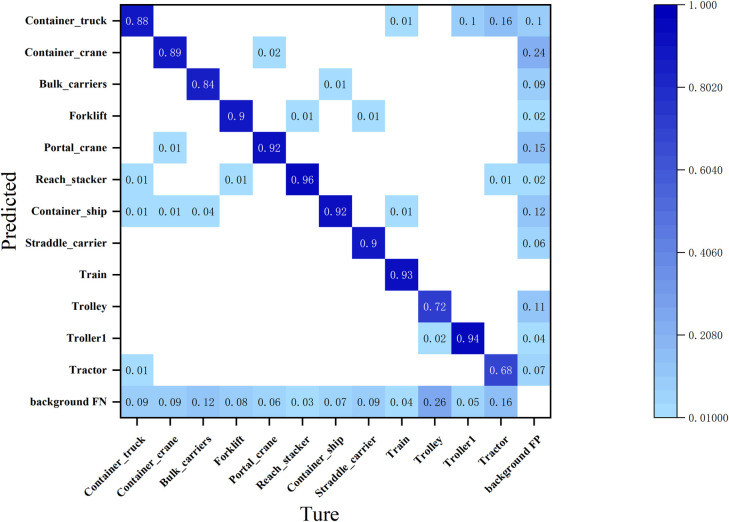
Confusion matrix for the improved models.

As illustrated in Fig 12, the misclassification rate of Bulk Carriers as Container Ships was 4.0%, while the misclassification rate of Container Ships as Bulk Carriers was 1.0%. This suggests that bulk carriers are more frequently misidentified as container ships within the terminal target dataset. Notably, the misclassification rate of container trucks as tractor cars is 3.0%, whereas the misclassification rate of tractor cars as container trucks is significantly higher at 16.0%. However, as shown in Fig 13 this latter rate decreases by 5.0% following model enhancements. This improvement indicates that the refined model exhibits an enhanced capability for fine-grained differentiation between terminal targets with similar appearances.

**Fig 14:**
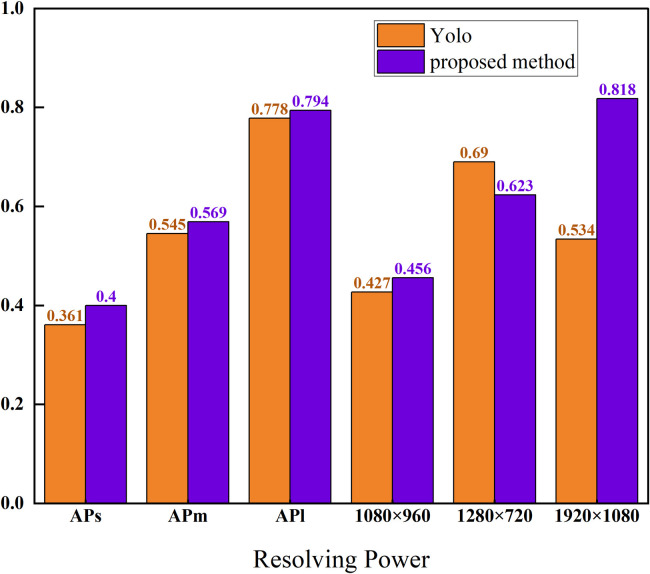
MAP of dock-site surveillance videos with different object sizes and resolutions.

To assess the impact of camera resolution and varying object sizes in surveillance footage at the marina project site, the proposed methodology was evaluated using 1200 surveillance images captured at the three most prevalent resolutions. Fig 14 illustrates the mAP of the surveillance videos from the terminal site across different object sizes and resolutions, demonstrating that the proposed method attains the optimal AP. In comparison to the original YOLO model, the proposed model demonstrated performance improvements of 3.9%, 2.4% and 1.6% in average precision (AP) for detecting small (size < 322), medium (size < 962) and large (size > 962) objects, respectively. The proposed method exhibited the highest accuracy in detecting large objects and was evaluated using monitoring video images of various resolutions. The results indicate that at a resolution of 1280 × 720, the AP of the model is marginally lower compared to YOLOv5s. However, at other resolutions, the model’s AP is substantially higher than that of YOLOv5s. This suggests that the overall performance of the model is superior.

### 4.2 Evaluation of overall structural results

#### (i) Model testing.

To comprehensively assess the tracking algorithm, the trained feature extraction network was integrated with both the SORT algorithm and the YOLOv5s algorithm, which was trained on the COCO dataset. Subsequently, the tracking performance of the algorithms, utilizing both the original and the enhanced networks, was evaluated on the MOT16 dataset with a focus on pedestrian targets. The outcomes of these evaluations are presented in Table 3. The tracking algorithm of the enhanced feature extraction network demonstrates a modest improvement of 0.5% in MOTA, alongside a reduction of 46 IDSWs, equating to a 12.5% decrease in switching occurrences. This reduction signifies enhanced tracking stability. Additionally, the metrics IDF1, IDR and IDP show improvements of 1.5%, 1.2% and 2.2%, respectively, indicating that the tracking efficacy of both the original and improved networks surpasses that of the original algorithm.

**Table 3:** Comparison of model testing results on the MOT16 dataset.

Methodologies	Datasets	MOTA	MOTP	IDSW	IDR	IDP
Raw Feature Extraction Networks	MOT16-02	20.7%	80.0%	59	18.5%	67.0%
	MOT16-04	32.9%	81.0%	70	30.7%	78.5%
	MOT16-05	48.4%	74.3%	65	51.3%	78.3%
	MOT16-09	53.9%	79.2%	44	46.9%	69.8%
	MOT16-10	37.0%	74.8%	63	32.2%	64.6%
	MOT16-11	53.2%	82.8%	24	49.4%	69.9%
	MOT16-13	19.4%	68.8%	42	22.3%	65.5%
	MOT16-train	33.6%	78.7%	367	31.6%	72.7%
Improved feature extraction network	MOT16-02	20.9%	79.9%	50	19.6%	70.2%
	MOT16-04	33.0%	81.0%	63	30.9%	79.0%
	MOT16-05	48.6%	74.3%	70	51.8%	79.0%
	MOT16-09	58.6%	79.6%	38	51.4%	71.7%
	MOT16-10	37.2%	74.8%	53	34.3%	68.3%
	MOT16-11	53.4%	82.8%	16	53.8%	75.9%
	MOT16-13	20.0%	68.7%	31	23.3%	68.1%
	MOT16-train	34.1%	78.8%	321	32.8%	74.9%

#### (ii) Validation of results.

**Fig 15:**
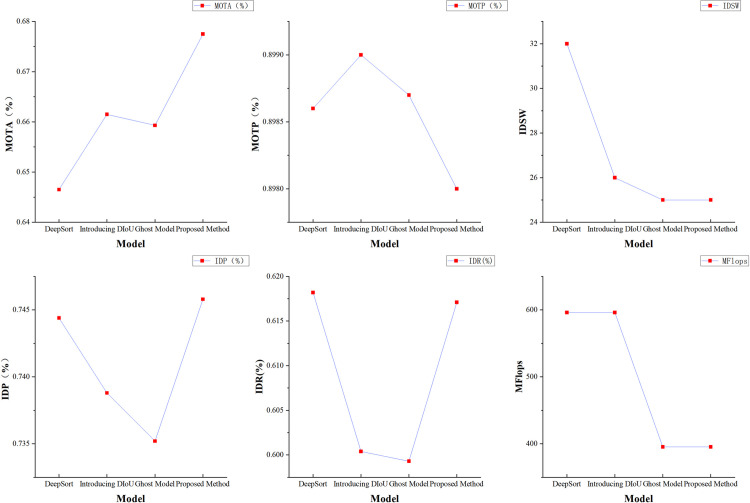
Indicator results of the improvement module.

During the model testing phase, the YOLOv5s model was employed as the detector for dock-moving machinery. The model was subsequently evaluated using both the original and improved algorithms, with the results presented in Fig 15. The MOTA for dock machinery, based on the DeepSORT algorithm, achieved a value of 65.65%, while the MOTP was recorded at 89.86%. Additionally, the number of tracking ID changes was observed to be 32. The experiments substantiate the outcomes of the DeepSORT algorithm. Validation demonstrates that the Ghost module-based epistemic feature extraction network effectively reduces computational complexity by 33% without compromising accuracy. Additionally, the number of tracking ID changes has decreased to 25. These findings suggest that the Ghost module-based feature extraction network enhances the tracking performance of moving targets in dock environments. When the DIoU metric is employed as the matching criterion between the prediction frame and the detection frame, there is a slight improvement in the MOTA compared to the MOTP. Additionally, the number of tracking ID changes decreases to 26. This finding suggests that the DIoU metric, which takes into account the distance from the center point, can be advantageous in reducing the frequency of tracking ID changes for dockside targets relative to the original metric. After comparing the original algorithm and this paper’s method, the experimental results show that constructing the feature extraction network through the Ghost module and combining with the DIoU matching metrics help improve the target trajectory accuracy and tracking position prediction accuracy, the final MOTA reaches 67.75%, which is a 2.19% improvement, the number of tracking ID variations is reduced to 25, and the model floating-point computation is reduced by 33.67%, The experimental results demonstrate that the method proposed in this paper effectively reduces the computational complexity of the feature extraction network while enhancing the accuracy of tracking moving targets on the dock.

To evaluate the efficacy of the tracking algorithm presented in this paper when combined with various detectors, the metrics of the improved detector model were tested using the mechanical tracking dataset. The results of these tests are presented in Table 4.

**Table 4:** Overall metrics results.

Model	MOTA	MOTP	IDSW	IDP	IDR
YOLOv5+DeepSORT	65.46%	89.86%	32	74.44%	61.82%
YOLOv5+proposed method	67.75%	89.80%	25	74.58%	61.77%
Improved YOLOv5+DeepSORT	70.26%	89.97%	18	76.61%	67.46%

Based on the test results of various algorithms applied to dockside mobile machinery, it is evident that the improved YOLOv5s, when combined with the enhanced DeepSORT tracking method, achieves MOTA, IDSW, IDP, and IDR metrics of 70.26%, 18, 76.61% and 67.46%, respectively. Initially, by solely enhancing the tracking algorithm, the model exhibited a 2.19% improvement in MOTA metrics, while the IDSW metrics decreased to 25. Subsequently, under the same tracking algorithm conditions, the improved YOLOv5s demonstrated a further 2.51% enhancement in MOTA metrics.

**Fig 16:**
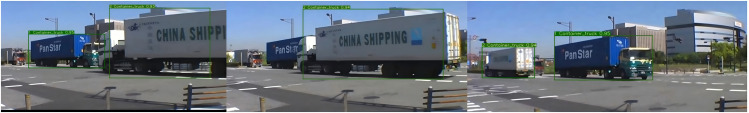
The tracking effect of container trucks in the port scenes.

**Fig 17:**
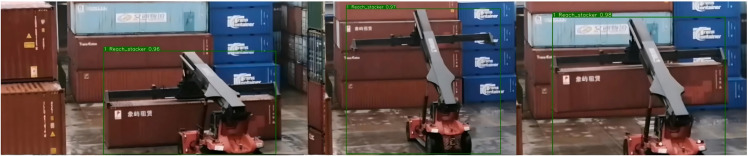
The tracking effect of reach stacker in the port scene.

**Fig 18:**
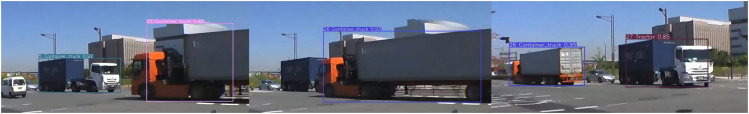
The tracking effect of container trucks in the port scenes.

**Fig 19:**
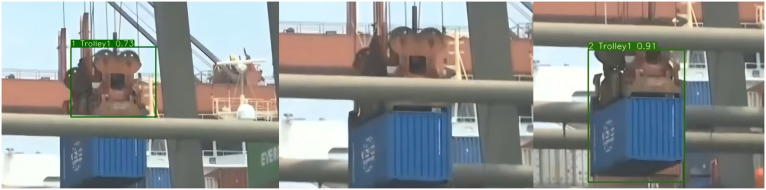
The tracking effect of trolley1 in the port sceneConclusion.

Overall, the enhanced detector, in conjunction with the optimized tracking algorithm, achieved a 4.8% increase in tracking accuracy compared to the original method. Additionally, the IDSW was reduced by 44%, indicating a more stable tracking performance. The improved method also achieved gains of 2.23% in the IDP metric and 5.64% in the IDR metric, further substantiating the overall enhancement in tracking efficacy as presented in this study.

(iii) Analysis of Tracking Effect in Port SceneAs presented in Figs 16 and 17, container trucks are exemplary,when a fleeting and complete occlusion transpires between container trucks bearing IDs 1 and 2 and container truck 1 disappears from the frame, after the vanishing of the occlusion, the tracker can still seamlessly match container truck 1 based on the detector without reassigning the ID. Similarly, when the trolley of the quay crane experiences local oclusion because of the obstruction by the boom during the container lifting process, and the detector remains competent to predict the bounding box, the tracker can still accurately match the ID. As demonstrated in Figs 18 and 19 , other metrics will regenerate matching IDs when occlusion takes place during the movement of the target, and the precision is relatively low. Hence, for the situations in the port scene, the function of DIOU matching in the appearance feature extraction link of DeepSORT surfaces, since the similarity of the appearance features significantly determines the success of the matching when re-matching occurs after the disappearance of the detection box.

## 5. Conclusion

In this study, an enhanced YOLOv5s integrated with the Deep SORT algorithm was proposed for the recognition and tracking of harbor terminal targets. This approach aims to facilitate early warnings regarding the efficiency of harbor terminal operations and to identify unexpected situations. The primary findings of this research are as follows:

(I) The introduction of multi-scale convolution enhances the granularity of the convolution kernel and effectively improves its ability to extract features across different scales; Combined with the pyramid segmentation attention mechanism and utilizing multi-scale attention within the feature vector channel, the detection accuracy is significantly improved. Compared with other models, the enhanced model has recognition accuracies of 99.3%, 96.5%, and 96.3% for port trains, container forklifts, and container front forklifts, respectively.
(II) Building convolutional layers with wide residual modules in feature extraction networks based on Ghost modules effectively reduces the number of floating-point operations; Introducing the DIoU index to merge centroid distances during the matching phase enhances the alignment between predicted and detected frames, improving the rationality of the matching process. The performance indicators are as follows: MOTA is 67.75%, MOTP is 89.80%, IDSW is 25, IDP is 25, IDR is 89.80%, MFlops is 395.41.
(III) Improved YOLOv5s+DeepSORT port terminal mobile target recognition and tracking algorithm, compared with the original method, has improved tracking accuracy by 4.8%, reduced IDSW by 44%, and increased IDP and IDR by 2.23% and 5.64% respectively. This algorithm can achieve more efficient and stable tracking of multiple moving targets in terminal scenarios, meeting the application requirements of multi-target recognition and monitoring in terminal environments.
